# Executive protocol designed for new review study called: systematic review and artificial intelligence network meta-analysis (RAIN) with the first application for COVID-19

**DOI:** 10.1093/biomethods/bpac038

**Published:** 2023-01-17

**Authors:** Nader Salari, Shamarina Shohaimi, Aliakbar Kiaei, Amin Hosseinian-Far, Kamran Mansouri, Arash Ahmadi, Masoud Mohammadi

**Affiliations:** Department of Biostatistics, School of Health, Kermanshah University of Medical Sciences, Kermanshah, Iran; Department of Biology, Faculty of Science, University Putra Malaysia, Serdang, Selangor, Malaysia; Department of Computer Engineering, Sharif University of Technology, Tehran, Iran; Department of Business Systems & Operations, University of Northampton, Northampton, UK; Medical Biology Research Centre, Kermanshah University of Medical Sciences, Kermanshah, Iran; Department of Electrical and Computer Engineering, Faculty of Engineering, University of Windsor, Windsor, Canada; Cellular and Molecular Research Center, Gerash University of Medical Sciences, Gerash, Iran

**Keywords:** artificial intelligence, systematic review, meta-analysis, network meta-analysis, protocol, COVID-19

## Abstract

Artificial intelligence (AI) as a suite of technologies can complement systematic review and meta-analysis studies and answer questions that cannot be typically answered using traditional review protocols and reporting methods. The purpose of this protocol is to introduce a new protocol to complete systematic review and meta-analysis studies.

In this work, systematic review, meta-analysis, and meta-analysis network based on selected AI technique, and for *P* < 0.05 are followed, with a view to responding to questions and challenges that the global population is facing in light of the COVID-19 pandemic.

Finally, it is expected that conducting reviews by following the proposed protocol can provide suitable answers to some of the research questions raised due to COVID-19.

## Background

The increase in the number of publications in many fields leads to the accumulation of results of these publications. One of the fundamental methods in research is to gather all existing published results and to provide a set of collective findings; this denotes the notion of “review” studies [1]. Nowadays, several researchers are finding systematic reviews for a helpful start in conducting their research, since systematic reviews outline what has been accomplished, and what could be the new trajectory for research. Moreover, in such review studies, several previous pieces of research are pulled together in one place and have become more accessible. Accordingly, by combining the results of the gathered studies through meta-analyses, an accumulated collective set of results are approximated. Due to the benefits offered, there seems to be a growing trend in the number of systematic reviews and meta-analyses research works [1, 2].

Systematic reviews usually have a very specific focus based on which an appropriate study question is identified. If there is a reasonable number of identified and collected studies with the same focus, meta-analyses techniques could be applied to amalgamate and combine all the results. Within health and medical studies, this type of research provides health policymakers with valuable insights that can assist with decision making [1, 2].

However, these studies do not always answer the designed questions, especially when there are too many studies in the subject area, leading to challenges in study identification, collection, and sieving. Moreover, in some instances, the collected studies entail a high heterogeneity in the way the original research works were conducted, and this in practice makes the answers to the review questions uncertain [3–5].

AI systems are systems that can demonstrate similar interactions to human intelligence including comprehending complex conditions, simulation of the thinking process and human inference, learning information, and inferring based on them [6, 7] and the progress of knowledge in the medical and the complexity of decision on screening, diagnosis, and treatment are bringing the experts’ attention toward using AI systems [6–8]. Machine learning (ML) is a branch of AI that focuses on learning and inference from data [6–8]. ML approaches are divided into two groups: supervised and unsupervised learning. In supervised learning, a collection of data with their corresponding category are used to train a model, while in the unsupervised approach, the category is not given [9].

The use of artificial intelligence (AI) has been reported in various studies to help medical science, Ahlrichs *et al*. [10] in a review study reported high efficiency of AI algorithms in the diagnosis of Parkinson’s disease. In a study by Bozkurt *et al*., the authors used electrocardiography of 10 patients with obstructive sleep apnea (OSA) against 10 healthy controls. This study first extracted the heart rate variability (HRV) from electrocardiograms (ECG), then extracted the QRS component at different frequencies using a digital filter, and then selected the feature using principal component analysis. The classification was performed by the *k*-nearest neighbors’ algorithm [11].

One type of recent study of drugs and their effects is network meta-analysis. Although this study is about drugs, it has changed the normal course of routine systematic review and meta-analysis studies. Instead of focusing on one specific question, it answers several questions [12]. Nonetheless, if we have a very high number of articles in a given period of time yet require reliable information, the traditional systematic reviews are unable to provide an answer to the posed question. Suppose we intend to determine whether we could find a cure for incurable cancer by collecting all the articles about that cancer? For instance, by suggesting a diet or by identifying a target group to control and reduce disease complications

Perhaps, we can look at a more topical research challenge, the COVID-19 pandemic, which since its inception in December 2019 until December 2020 has swept across the world, causing more than 80 million infections and 1.5 million mortalities. The pandemic has also resulted in fear, stress, and other negative psychological, social, and economic damages globally [13–16]. What is certain is that the efforts of the international community and researchers to combat the disease are by determining the target group of the disease, prevention methods, diagnosis approaches, treatment measures and suitable vaccines, and several research questions associated with each of these themes. By conducting a search in PubMed, from December 2019 to December 2020, over 82 000 COVID-19-related articles have been published, yet we are still helpless in answering the key research questions. For instance, we are still uncertain about how the prevention methods should be implemented globally, and in different countries. We still do not know with absolute certainty, if quarantine is the same or different from isolation, and if so, where and when should it be used? Similarly, we do not know what drug combination or diet is useful for treating patients? We are unable to conclude whether traditional medicine and common medical treatments can be used in the treatment of this disease or not? We still do not know if children show the same symptoms as adults if infected? Although these and other questions vary across regions, populations and enmities, age groups, and genders, such questions need to be answered as COVID-19 is still spreading and taking lives.

We have used a combination method based on the weighted version of the power mean in the transformed distribution. We have proposed an ML method that uses *P*-values between COVID-19 and affected human genes, as well as *P*-values between those genes and drug associations or diet associations or sex and age associations, or another question as input. The output is finding associations of cooperation that make a small combined *P*-value with COVID-19. This algorithm computes all combined *P*-values between association drugs and COVID-19, and these steps are iterated until that significance becomes less than a threshold. While doing and writing the protocol in order to be practical, we have our method to answer the question of what is the best drug treatment in the treatment of patients with COVID-19? We have used this method and answered this question with AI and the Rhine method [17].

Therefore, in this article, we would like to outline a new protocol that can streamline the systematic review process, in particular in contexts where traditional systematic reviews are unable to provide a mechanism for identifying and collecting relevant studies. Additionally, we demonstrate how the new protocol can assist with finding responses to questions such as the ones provided in the next section.

## Materials and methods

### Protocol and registration

The research protocol was registered in the PROSPERO international prospective register of systematic reviews (CRD42021256797).

### Questions

1. Who are the target population of COVID-19?
2. What are the definitive symptoms of COVID-19 patients?
3. What is the definitive prevention method for COVID-19?
4. What is the definitive method for detecting COVID-19?
5. What is the definitive drug regimen for the treatment of patients with COVID-19?
6. What is the preventive diet for the population without COVID-19?
7. What is the diet that helps in the treatment of patients with COVID-19?
8. Who are the key scientists who have had promising results in responding to COVID-19?
9. What are the symptoms (such as Respiratory distress, Anosmia and Ageusia) that may indicate a higher probability of having COVID-19 among the infected population?
10. What procedures (such as quarantine, convalescent plasma, reverse genetics, phylogenetic analysis, total protein measurement, etc.) can have a better effect on the treatment of COVID-19?
11. Which body parts are affected the most when infected with COVID-19 (i.e. chest, throat swab sample, or respiratory system)?
12. What are the most common similar diseases (such as coronavirus infections, pneumonia, viral, severe acute respiratory syndrome)?
13. Which genes/proteins (e.g. ACE2, TMPRSS2, CDSN) are most associated with the disease?
14. What keywords does Medical Subject Headings (MeSH) include about COVID-19 (e.g. pandemics, beta-coronavirus, coronavirus)?
15. What are the chemicals associated with COVID-19 (such as angiotensin-converting enzyme 2, angiotensin-converting enzyme 2, spike glycoprotein, coronavirus)?
16. What are the cellular components (e.g. viral nucleocapsid, host cell) in COVID-19?
17. What are the biological processes of COVID-19 (e.g. transmission of the virus, viral release from host cell)?
18. What are the molecular functions of COVID-19 (such as receptor binding, ubiquitin-like protein binding, and trialkyl sulfonium hydrolase activity)?
19. What are the possible cell lines in COVID-19 (e.g. HeLa, MCF-7, K-562, Calu-3)?
20. Apart from modern medical sciences, are solutions based on traditional methods such as traditional Chinese medicine (such as ling mao xiang, shi wei, xiang hei zhong cao zi) suitable for the treatment of COVID-19?
21. How COVID-19 is classified according to MeSH?

These questions are considered as primary questions for researchers to use the new method, and therefore these questions can be expanded or modified according to the needs of the scientific community and other researchers.

### Collaboration

Methodology and method of work: M.M., Dr A.A.K., Dr N.S., Dr A.H.-F.

Statistical analysis and tests: M.M., Dr N.S.

AI: Dr A.A.K., Dr A.H.-F.

Conceptualization and analysis: M.M., Dr A.A.K., Dr N.S., Dr A.H.-F., Dr A.A., Dr K.M.

Relevant articles based on the proposed protocol will be conducted and published by the above researchers and will be published in the near future. The group of researchers can be expanded by accepting the opinions of researchers from other countries.

## Part 1: systematic review

The systematic review section will be based on Cochrane’s 7-step approach for searching and selecting studies. The steps include selecting a research question, determining inclusion and exclusion criteria, identifying articles, selecting studies, evaluating the quality of studies, extracting data, analyzing, and interpreting findings.

Moreover, the research question(s) and keywords determination will be conducted according to the population, intervention, control, and outcomes (PICO) guidelines.

Considering the research question as previously mentioned, and according to the PICO guidelines, the study population (Population), the intervention (Intervention), and comparison groups (Comparison) that can include the average scores of the indicators before and after the intervention is aligned with the outcome of the case in accordance with the study question (Outcome).

### Search for articles

Keywords will be extracted from the MeSH dictionary according to the PICO instructions. Keywords will be related to the study population (P), intervention (I), comparison (C), and outcome (O); these keywords will be outlined separately for each question.

### Article identification

In the proposed method, initially, an AI-based text mining approach conducts the searches within multitude of abstracts related to COVID-19 (e.g. genes, names of drugs, names of foods, etc.) to automatically rank the keywords related to COVID-19. Then, from these ranked keywords, it examines the top-ranked keywords in pairs. With this process, a large collection of initial papers is extracted that can be selected as input for the PICO phase.

**Figure 1.**
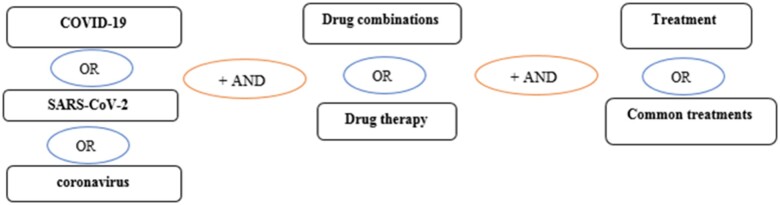
Determining search strategy using (AND) and (OR) operators in international databases.

Subsequently, to find studies related to the research question, the extracted articles are searched for compatibility within the international indices and databases such as ScienceDirect, Web of Science, ProQuest, Embase, Medline (PubMed), and Scopus. The lower and higher time limits for searching articles related to COVID-19 will be between 1 December 2019 and 31 December 2021. Given that English is the international language, the search process will look at articles published in English. Therefore, studies published in any other language will be excluded from the search and selection processes. The search strategy in each database will be determined through the Advanced Search feature, using all possible keyword combinations and with the help of ‘AND’ and ‘OR’ operators. The simple schematic view of this process is outlined in Fig. 1 (Fig. 1 shows an example of how to search for drugs combination in the treatment of COVID-19, and this example can be used for other questions and problems).

### Selection of studies based on inclusion and exclusion criteria

In order to access all relevant studies, the sources of articles that met the inclusion criteria will be manually reviewed. To avoid errors, all steps of the search process, study selection, quality evaluation, and data extraction will be performed independently by three reviewers. If there is a disagreement among the reviewers in relation to the exclusion or inclusion of an article, then a third reviewer will make the assessment to eliminate any sources of bias.

The information of all articles found in each database will be transferred into the EndNote X8 reference management software. After completing the search in all databases, duplicate articles will be deleted. At that time, in order to avoid the risk of subjectivity in the study selection process, the names of the authors and the titles of the journals of the articles will be removed, and a checklist will be prepared based on the title and abstract of the studies. Studies in which full text are not found and do not meet the inclusion criteria will be excluded from the systematic review process. The full text of all the remaining articles will be then evaluated [15, 16].

### Inclusion and exclusion criteria according to the research question

The inclusion criteria for article selection will be determined and stated in accordance with the type of the posed question and the purpose of the systematic review. The criteria for inclusion can be, for instance, clinical trials, cohorts, case–control, descriptive studies, etc. Similarly, considering the research question and the focus of the review, exclusion criteria will be determined and stated.

### Quality evaluation of studies

In order to evaluate the quality of the studies that are going to be selected for the review process, the Consolidated Standards of Reporting Trials (CONSORT) checklist will be used. CONSORT includes 25 general sections, with each section having sub-sections resulting in a total of 37 sub-sections. The various sections of this checklist include the title and abstract, introduction and context, methods, participants, interventions, outcomes, sample size, randomization, recruitment, statistical methods, etc. In order to rate the articles, if each article referred to the items considered in the checklist, it was given a score of 1 and if it was not mentioned, a score of zero was given. The minimum and maximum scores in this checklist are 0 and 37, respectively. Studies with 75% or more of the maximum achievable score (score greater than or equal to 27) with “high quality”, studies with a score between 75–50% (score 18–26) as “average quality”, and studies with a score lower than 50% (score less than or equal to 17) were considered as “low-quality” studies [18].

The Strengthening the Reporting of Observational Studies in Epidemiology checklist will be used to review observational studies, that is cohort, case–control, and cross-sectional. This checklist consists of 22 sections, 18 of which are general and applicable to all observational studies (cohort, case–control, and cross-sectional). The remaining sections are more focused on criteria. The criteria used on the checklist include study objectives, sample size, type of study, sampling method, research community, data collection method, definition of variables, data collection methods, statistical tests, and findings. In order to rate the articles, in this checklist, the maximum quality review score of 32 will be considered and articles with a score less than 14 will be considered to be of low quality and will be therefore excluded from the systematic review [14, 16].

The Newcastle–Ottawa Scale (NOS) checklist will also be used, when applicable. The NOS is a similar quality assessment method for observational studies that are recommended within the Cochrane guidelines [19].

### Study selection

**Figure 2.**
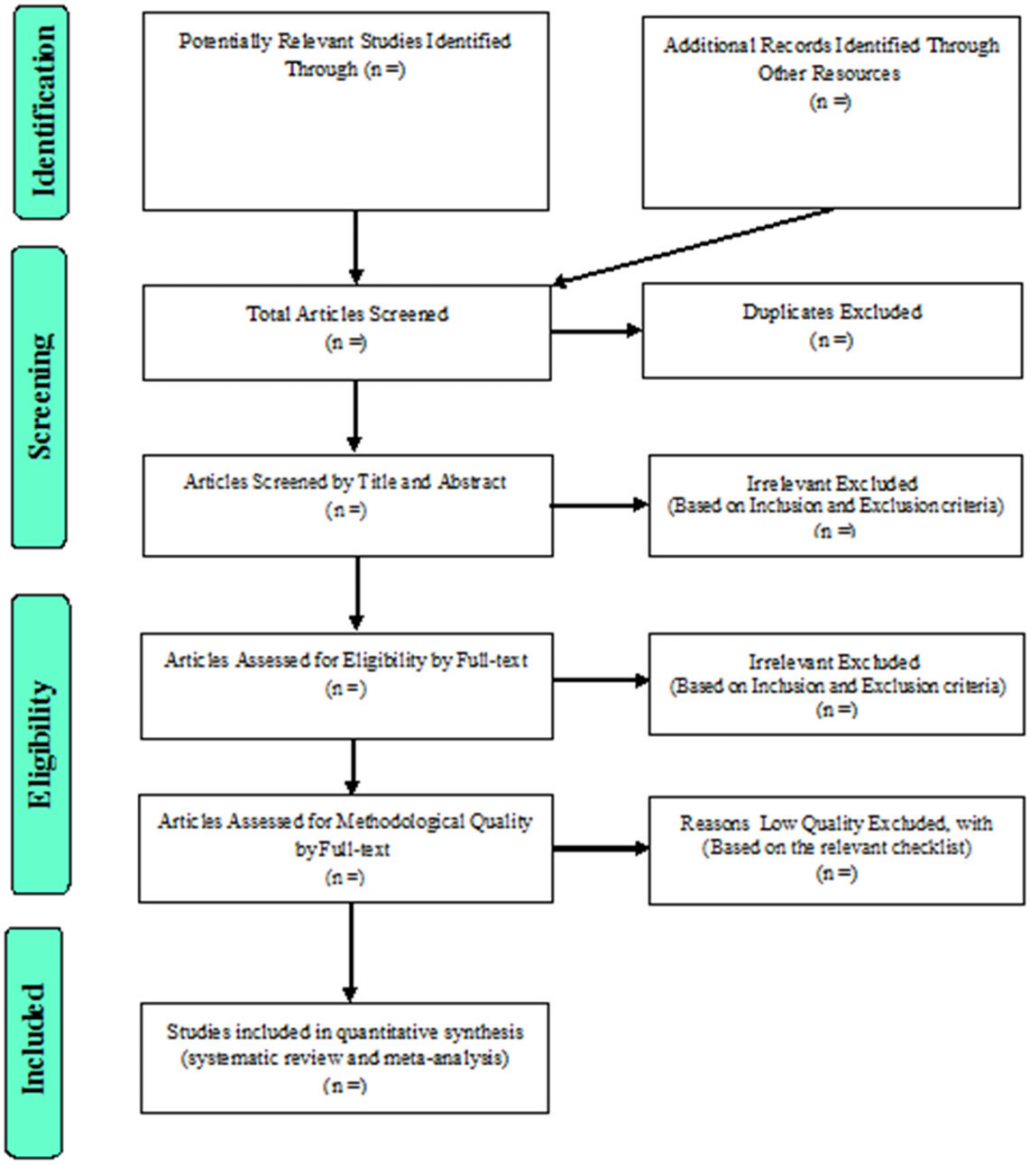
The flowchart on the stages of including the studies in the systematic review and meta-analysis (PRISMA 2009).

After the study selection in the systematic review and in order to select studies for meta-analysis, the four-step Preferred Reporting Items for Systematic Reviews and Meta-Analyses 2009 process, that is article identification, screening, eligibility evaluation, and finally study selection for meta-analysis will be followed (Fig. 2).

## Part 2: meta-analysis

### Statistical analysis

Based on the type of study and its quantitative information, that is relative risk, odds ratio, and mean scores, the method of analysis will be examined. To assess heterogeneity among studies, the *I*^2^ test will be adopted. We will use ROB tool to check the risk of bias of studies in our evaluation. To assess publication bias, Funnel plots and Egger’s test at the significance level of 0.05 will be followed. If the sample size is high among the collected studies, the Begg and Mazumdar rank correlation test will be used at the significance level of 0.1. The data will be analyzed within the Comprehensive Meta-Analysis software (version 3), and the significance level of the test will be considered *P*˂0.05 [16–18].

## Part 3: AI-based meta-analysis network

In this review, although three researchers create good reliability, some related articles may be forgotten or lost due to carelessness, fatigue, and neglect, so we use AI to carry out the review process, and AI performs an independent evaluation and completes the search process. In the usual systematic review studies, the search and extraction work is done by three independent researchers in order to increase the reliability of the study, and practically all the studies related to the subject are extracted from the reviewed databases, we also use AI in this new method as an independent researcher to conduct a search independent of human search in the investigated databases using the keywords designed for it. And it actually confirms the human search and points out the things that have been forgotten or lost so that they can be checked by a human researcher.

In addition, in analyzing the analysis, the AI looks for the lowest *P*-value created in the searched studies and reports the best and most complete analysis, and this also complements the human analysis.

**Figure 3.**
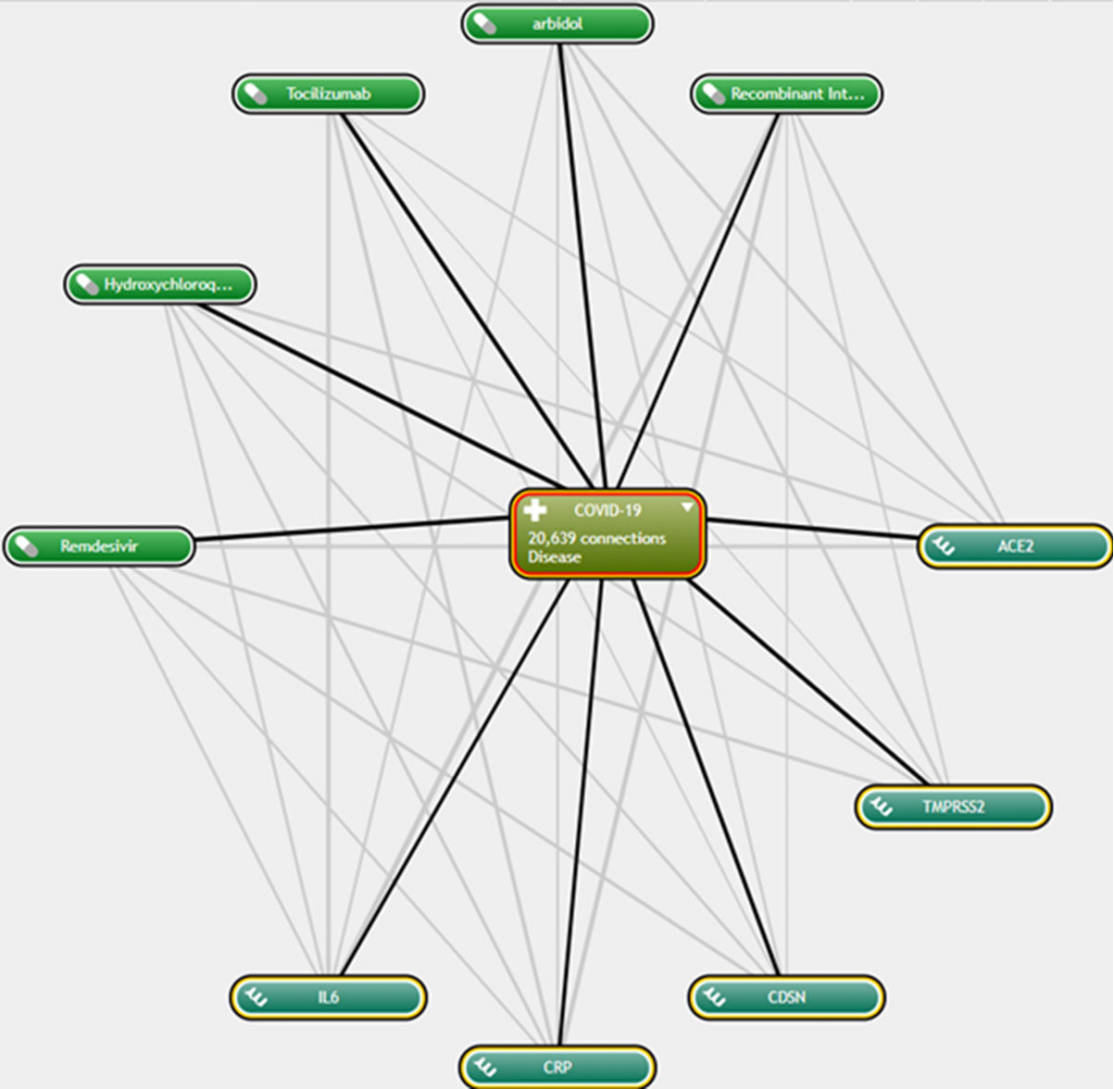
An example of the weighting of indicators examined by AI.

In this section, AI first forms a network graph between each of the components of COVID-19 (such as its genes) and the drugs, using text mining. The weight of each edge represents the *P*-value criterion in the network. The following figure shows an example of this network (Fig. 3).

**Figure 4.**
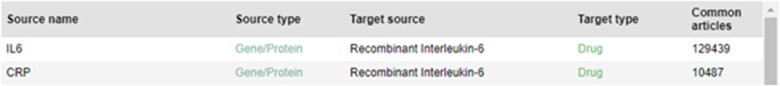
An example of the ranking of indicators examined by AI.

Then a ranking is made between each drug and each gene. This is shown in the figure below (Fig. 4):

Finally, the AI-based algorithm can approximate the best drug combination with the aim of having the greatest effect on the entire COVID-19 genome. To do this, the following steps apply:

1. The impact weight of each gene is calculated in COVID-19.
2. The drug from the above graphic network and the weight of each gene that has the most destructive effect on the entire COVID-19 genome are selected.
3. The destructive effect of the selected drug on the whole genome is calculated, and the effect weights of each gene are updated.
4. Steps 2 and 3 are repeated until the stop condition is met.

The stop condition in this experiment is to reach a *P* <0.05 of the threshold value. In other words, more drug is added to the drug composition so that the selected combination of drugs reaches the *P* <0.05 to be effective for the treatment of COVID-19.

## Discussion

Systematic review and meta-analysis studies are at the first rank of the pyramid of scientific evidence, and their information is very efficient for health policy-making and interventions, this information provides accurate evidence of disease status, prevalence, incidence, relationships, and inputs even more than clinical trial studies. Now, if the level of accuracy of evidence increases by adding AI, the common errors and distortions of systematic review and meta-analysis are reduced, and all studies are reviewed, then the amount of error in the decisions of health policy-makers at the community level will be greatly reduced.

In summary, the research process is expected to be as follows:

Step 1: Find all the *P*-values associated with COVID-19 genes and drugs.

Step 2: Find the combined *P*-value between the COVID-19 genome and each drug.

Step 3: Find some scenarios of the best drug combination for COVID-19 using the AI algorithm.

Step 4: Find related articles and delete unrelated articles through the systematic review.

Step 5: Thematic categories of articles for specific groups with the help of a meta-analysis.

Step 6: Find the best drug combination for each group identified in the previous step, using AI and according to the network meta-analysis protocol.

This process is novel, not only because it approximates a drug combination but also because the above steps lead to the validation of the drug recommendations that AI claims. The process layout is designed so that the required inputs of each step are provided by its previous step.

In other words, in the first three steps, AI is responsible for finding the number of drugs to treat COVID-19 that are fed into the systematic review process.

In the fifth step, the information from the previous step is placed within the meta-analysis protocol. Meta-analysis then finds a connection with these different drugs and groups, which are fed by AI in the sixth step. Finally, according to each group, AI states the best scenario of the drug combination.

According to the reported process, it is expected that reviewing articles with the method of Systematic Review and AI Network Meta-analysis will be able to answer the questions mentioned in the field of COVID-19 which can be ultimately an effective step in providing efficient policy-making in the era of the pandemic.

## Data Availability

Datasets are available through the corresponding author upon reasonable request.
